# Identification of Hydroxysteroid Dehydrogenase Type 1 As a Potential Bladder Tumor Marker

**DOI:** 10.61186/ibj.4068

**Published:** 2024-01-29

**Authors:** Aida Albadawy, Mohammed Alqudaimi, Hanyue Cui, Xianghui Yan, Jing Sun, Ping Shi

**Affiliations:** 1 State Key Laboratory of Bioreactor Engineering, East China University of Science and Technology, Shanghai 200237, China;; 2 Qinghai Key Laboratory of Qinghai-Tibet Plateau Biological Resources, Northwest Institute of Plateau Biology, The Chinese Academy of Sciences, Xining 810001, China

**Keywords:** Biomarkers, HSD17B1, Survival

## Abstract

**Background:**

The HSD17B family has been implicated in the prognosis and treatment prediction of various malignancies; however, its association with BLCA remains unclear. This study aimed to evaluate the potential of *HSD17B1*, as a prognostic biomarker, for the survival of patients with BLCA and to determine its effectiveness as a supplemental biomarker for BLCA.

**Methods:**

A series of bioinformatics techniques were applied to investigate the expression of *HSD17B1* in different types of cancer and its potential association with the prognosis of BLCA patients using diverse databases. The UALCAN, Human Protein Atlas, cBioPortal, Metascape, GEPIA, MethSurv, and TIMER were employed to analyze expression differences, mutation status, enrichment analysis, overall survival, methylation, and immune-infiltrating cells. The qRT-PCR was implemented to detect the mRNA expression levels of *HSD17B1* in vitro.

**Results:**

Elevated mRNA and protein levels of *HSD17B1*, surpassing normal levels, were observed in BLCA samples. In addition, the BLCA patients with higher mRNA expression level of *HSD17B1* significantly reduced the OS. Also, several immune infiltrating cells, including mast cell resting CIBERSORT-ABS, have been identified as tumor-associated biomarker genes, with the potential to significantly influence the immunological environment. Finally, qRT-PCR analysis revealed a significant upregulation of *HSD17B1* mRNA expression level in the cancer cells compared to the human 293T cells, which was consistent with the bioinformatics data.

**Conclusion:**

There is a strong correlation between the elevated *HSD17B1* expression and positive prognosis in patients with BLCA. Therefore, *HSD17B1* can be used as a prognostic biomarker in these patients.

## INTRODUCTION

Bladder cancer is a leading cause of mortality among malignant diseases and associated with a high incidence rate. Tumors at early stage demonstrate minimal malignant potential and are often associated with lower cancer progression and mortality rates^[^^1^^]^; however, they have a poor prognosis. Therefore, early detection and effective treatment of cancer are crucial for increasing the overall survival rates of the patients^[^^2^^,^^3^^]^. Considering these data, the improvement and development of BLCA therapeutics have recently gained the interest of researchers. 

The 17HSDs are enzymes that regulate the levels of biologically active estrogens and androgens. To date, 14 distinct types of these enzymes have been identified. Each type is designated based on its roles in the activation of 17-keto and 17-hydroxysteroids, either by reduction or oxidation of the carbon^[^^4^^]^. These processes involve biological reactions that rely on either NAD^+^/NADH or NADP^+^/NADPH^[^^5^^]^. The HSD17B enzyme has been discovered to be a potential biomarker for BLCA patients^[^^6^^]^. Alterations in *HSD17B1* expression are related to various hormone-dependent disorders, including breast cancer, endometriosis, endometrial hyperplasia cancer, and ovarian epithelial cancer. *HSD17B1* plays a significant role in converting less active estrogen (E1) to considerably potent estradiol (E2)^[^^7^^]^. However, it remains unclear whether the expression of this enzyme is associated with BLCA.

In the current study, using bioinformatic techniques, we evaluated the prognostic and diagnostic significance of *HSD17B1* in BLCA. Subsequently, we identified its expression in various cell lines by qRT-PCR.

## MATERIALS AND METHODS

**Examining the level of human** ***HSD17B1*** **mRNA expression in different cancers**

The UALCAN website, available at http://ualcan. path.uab.edu/, serves as a valuable online resource offering comprehensive, user-friendly, and interactive information. The website was created using PERL CGI and features high-quality graphics with JavaScript and CSS. This tool can effectively utilize and extract publicly accessible cancer OMICS data from various sources, including TCGA, MET500, CPTAC, and CBTTC. It also provides graphs and plots regarding analysis of genes of interest^[^^8^^]^. In the current study, we utilized UALCAN to investigate the mRNA expression of *HSD17B1* in BLCA patients and evaluate their potential association with clinicopathologic parameters, as well as to assess the differential expression of the *HSD17B1* gene between tumor and normal samples. Differences in gene expression were tested utilizing a student's t-test. Statistical significance was determined at a *p* < 0.05. 

**Determining** ***HSD17B1*** **protein expression level in BLCA**

The Human Protein Atlas database (https://www. proteinatlas.org) is an open online dataset that offers comprehensive information about human proteins in cells, tissues, and organs. This resource is valuable for researchers and scientists seeking a detailed information on the expression and localization of various proteins within the human body. Furthermore, it provides immunohistochemistry-derived expression data for around 20 common forms of cancer^[^^9^^]^. The database allows to easily compare the differential protein expression in genes of interest between malignant and normal tissues. In the present study, we applied immunohistochemistry images to assess the protein expression of *HSD17B1 *in both human normal and BLCA tissues.

**Investigating** ***HSD17B1*** **genetic alteration and survival rates in BLCA patients**

The primary objective of the cBioPortal is to facilitate the investigation of cancer genomics. This web-based platform is accessible at http://www.cbioportal.org and utilized for the analysis and exploration of cancer at the genetic level. The platform serves as a free tool for the interactive analysis of multidimensional genomic datasets associated with cancer. It offers quick and simple access to clinical features and molecular profiles earned from extensive cancer genomics initiatives, enabling rapid investigation and ensuring high data quality^[^^10^^]^. In this work, we employed cBioPortal to explore the genetic alteration of *HSD17B1* in BLCA and its association with the OS and DFS in BLCA patients. Both OS and DFS are important indicators used to evaluate the duration of a patient's survival after being diagnosed with BLCA. OS specifically measures the patient's OS rate, while DFS evaluates the period during which the patient remains free from the disease after receiving treatment. Overall, 474 samples from BLCA MSK-TCGA-2020 cBioportal were analyzed. The copy-number alteration data utilized in this study were gathered from the GISTIC. We applied a threshold of ± 1.8 to the mRNA expression z-scores to determine the importance of changes, which was carried out using the RNA Seq V2 RSEM method and compared to diploid samples.

**Analysis of functional enrichment in similar genes**

Metascape (http://metascape.org) is a web-based portal tool that can be used for comprehensive analysis and interpretation of OMICS-based studies. Metascape provides a one-click express analytical interface for producing interpretable results. The functions of this tool include gene annotation, interactome analysis, function enrichment, and membership search^[^^11^^]^. In this work, we operated GO and KEGG functional enrichment analyses on similar genes. A protein-protein interaction network was created using MCODE algorithm, and significant gene modules were screened. *P* < 0.01 was the significance cutoff, and the functions of similar genes and signal pathways enrichment were visualized through horizontal histograms.

**Investigation of** ***HSD17B1*** **mRNA expression level in BLCA patients**

The GEPIA web server, developed by Zhang's Lab at Peking University (Beijing, China), is a specialized platform for gene expression analysis. GEPIA utilizes data from both cancer and normal samples obtained from TCGA and the GTEX databases. This tool harnesses RNA-Seq data from the UCSC Xena project and offers customizable functions, including differential expression analysis between tumor and normal samples and the identification of the related genes. This resource is highly valuable for researchers who are interested in analyzing gene expression in the context of cancer and normal tissues^[^^12^^]^. In the current work, GEPIA was used to perform the correlative prognostic of gene and identify similar genes associated with *HSD17B1*. To demonstrate the association between *HSD17B1* and the prognosis of BLCA patients, we used survival curves (survival plot) and gene expression relative to tumor grade (stage plot) in GEPIA. We determined statistical significance by considering a *p* value of less than 0.05. In addition, we employed Kaplan-Meier plots to visually depict the survival outcomes of patients diagnosed with BLCA.

**Identification of the methylation role in the expression of** ***HSD17B1***

MethSurv (https://biit.cs.ut.ee/methsurv^[^^16^^]^) is an online platform that provides methylation analysis of biomarkers. Data from TCGA were used in this investigation. The web application framework used to create this particular item is called R Shiny. It was created especially for the R programming language. In studying DNA methylation, its levels are typically expressed as beta values, which can range from 0 to 1. These beta values can be measured by applying the following formula: β = M/(M + U + 100), where M and U denote the intensity of DNA methylation and unmethylation, respectively, and β is the measure of DNA methylation^[^^13^^]^. Herein, we used MethSurv to compare different CpG sites in BLCA samples.

**Studying the role of immune-infiltrating cells in *HSD17B1***

The TIMER (https://cistrome.shinyapps.io/timer/) was used to compare the expression levels of immune cell infiltration of the *HSD17B1* gene. The gene model was utilized to compare the extent of tumor infiltration in tumors with varying types of immune cells. In this study, we analyzed 41 different types of immune infiltration cells in the sample. We also used a cutoff value of *p* ≤ 0.05 to determine the significance of the relationship between BLCA cells and the host immune system.

**Cell culture**

In the present study, the samples of human urinary bladder carcinoma (UM-UC-3) cells, liver cancer (SMMC-2271), cervical cancer (HeLa), and human breast cancer (MCF-7) cell lines were obtained from the Chinese Academy of Science in Shanghai, China. The cells were cultured in DMEM media (Gibco/Invitrogen, Camarillo, CA, USA) containing 10% of fetal bovine serum (PAN-Biotech, Aidenbach, Germany). The cells were then maintained in an incubator with a temperature of 37 °C and an environment containing 5% carbon dioxide.

**qRT-PCR analysis**

To extract total RNA from the cells, we used the TRIeasy™ Total RNA Extraction Reagent, which was manufactured by Yeasen Biotechnology in Shanghai, China. The extracted RNA was reverse-transcribed into cDNA using the Hifair® II 1st Strand cDNA Synthesis Super Mix for qPCR (gDNA digester), produced by Yeasen Biotechnology. For the RT-qPCR experiment, we utilized the Hieff UNICON® qPCR SYBR Green Master Mix (Yeasen Biotechnology) as the reaction mixture. The experiment was conducted on a Bio-Rad CFX96 System (HERCULES, California, USA). The PCR cycle conditions were as follows: initial denaturation at 95 °C for 30 seconds, followed by 40 cycles, each consisting of 10 seconds at 95 °C and 30 seconds at 60 °C. The 2^-ΔΔCt^ relative quantification method was used to determine the relative expression levels of *HSD17B1* mRNA. The significance of the expression analysis was assessed using a student's t-test. Table 1 lists the primers used for qPCR (model genes).

## RESULTS

**Expression levels of human** ***HSD17B1*** **mRNA in different cancers**

**Fig.1:**
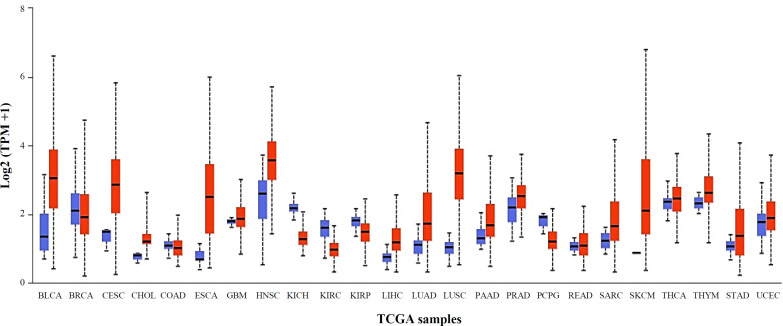
Transcriptional expressions of *HSD17B1* in different types of cancer (UALCAN database). Blue: normal; Red: tumor. Transcriptional expressions of *HSD17B1* in different cancer types were examined using the UALCAN database. BLCA: bladder cancer; BRCA: breast invasive carcinoma; CESC: cervical squamous cell carcinoma; CHOL: cholangiocarcinoma; COAD: colon adenocarcinoma; ESCA: esophageal carcinoma; GBM: glioblastoma multiforme; HNSC: head and neck squamous cell carcinoma; KICH: kidney chromophobe; KIRC: kidney renal clear cell carcinoma; KIRP: kidney renal papillary cell carcinoma; LIHC: liver hepatocellular carcinoma; LUAD: lung adenocarcinoma; LUSC: lung squamous cell carcinoma; PAAD: pancreatic adenocarcinoma; PRAD: prostate adenocarcinoma; PCPG: pheochromocytoma and paraganglioma; READ: rectum adenocarcinoma; SARC: sarcoma; SKCM: skin cutaneous melanoma; THCA: thyroid carcinoma; THYM: thymoma; STAD: stomach adenocarcinoma; UCEC: uterine corpus endometrial carcinoma

Using the UALCAN, the mRNA expression of *HSD17B1* was determined to investigate its expression in different cancer types. According to the findings (Fig. 1), *HSD17B1* exhibited significantly higher mRNA expression in various tumor samples than that of normal and primary samples. The tumor samples include lung squamous cell carcinoma, cervical squamous cell carcinoma, head and neck squamous carcinoma, thyroid carcinoma, BLCA, and stomach adeno carcinoma. The statistical analysis showed significant *p* values for *HSD17B1*, including values like *p* < 1E-12 and *p* = 1.62E-12 among lung squamous cell carcinoma and stomach adenocarcinoma. This result indicates that the transcriptional expressions of *HSD17B1* were significantly overexpressed in different types of cancer. 

**Table 1:** Primers used for qRT-PCR (model genes)

**Gene**	**Primer**	**Sequence (5'-3')**
*GAPDH*	Forward	GAGAAGGCTGGGGCTCATTT
	Reverse	AGTGATGGCATGGACTGTGG
*HSD17B1*	Forward	TGATGGGGCTGCCTTTCAAT
	Reverse	ACTCGATCAGGCTCAAGTGG

**Expression levels of** ***HSD17B1*** **protein in BLCA**

**Fig. 2:**
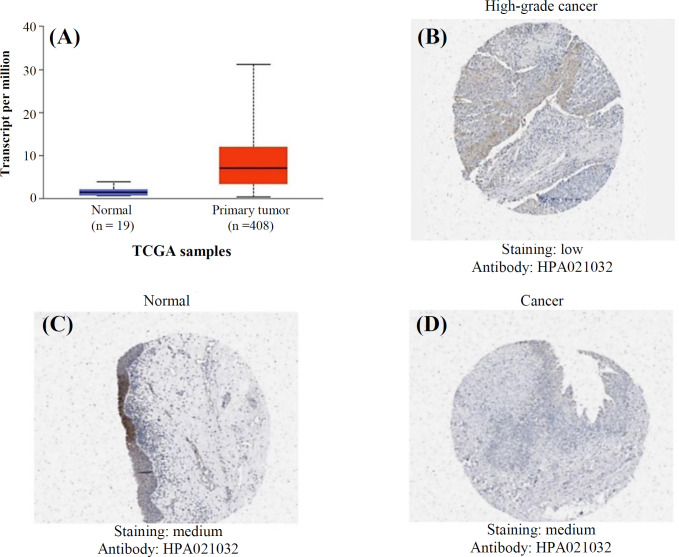
The mRNA and protein expression of *HSD17B1* in BLCA and normal urethral bladder tissue. (A) The mRNA expression of *HSD17B1* in BLCA tissue compared to normal samples using data from the UALCAN database *p* = 1.85E-05; (B-D) shows the immunohistochemistry images of HSD17B in normal bladder tissue and BLCA tissue (Human Protein Atlas)

Investigating the specific mRNA expressions of *HSD17B1* in bladder tumors showed a significant increase in the mRNA expression of *HSD17B1* in BLCA tissues compared to the normal sample (*p* = 1.85E-05; Fig. 2A). We utilized the human Protein Atlas database to assess the protein expression levels of *HSD17B1* in BLCA. Our findings indicated that the expression of the *HSD17B1* protein was moderate in normal BLCA, whereas it was medium to low in high-grade urothelial carcinoma patients' tissues (Fig. 2B). According to the findings, the levels of *HSD17B1* gene and protein expressions were significantly higher in BLCA patients than the normal sample (Fig. 2C and 2D).

**Clinicopathological relationship between** ***HSD17B1*** **mRNA levels and BLCA clinicopathological features patients**

BLCA patients exhibited elevated levels of *HSD17B1* mRNA and protein expression. Consequently, we employed UALCAN to analyze the clinicopathological features of BLCA to establish a correlation between *HSD17B1* mRNA levels and patient sample types. We found that the mRNA and protein levels of *HSD17B1* were markedly higher in cancer samples than normal samples in patients, with a statistically significant association of *p* = 1.85E-05 (Fig. 2). Additionally, we examined the correlation between the mRNA levels of *HSD17B1* and individual cancer stages and detected a significant association between the *HSD17B1* mRNA expression levels. Also, there was a significant association of *HSD17B1 *stages 2, 3, and 4 when compared to normal patients, with *p *values of 9.76E-03, 9.76E-03, and 9.76E-03, respectively. No statistically significant difference was found between cancer stage 1 and normal patients (Fig. S1). 

***HSD17B1*** **genetic alteration and survival outcomes in BLCA patients**

**Fig. 3:**
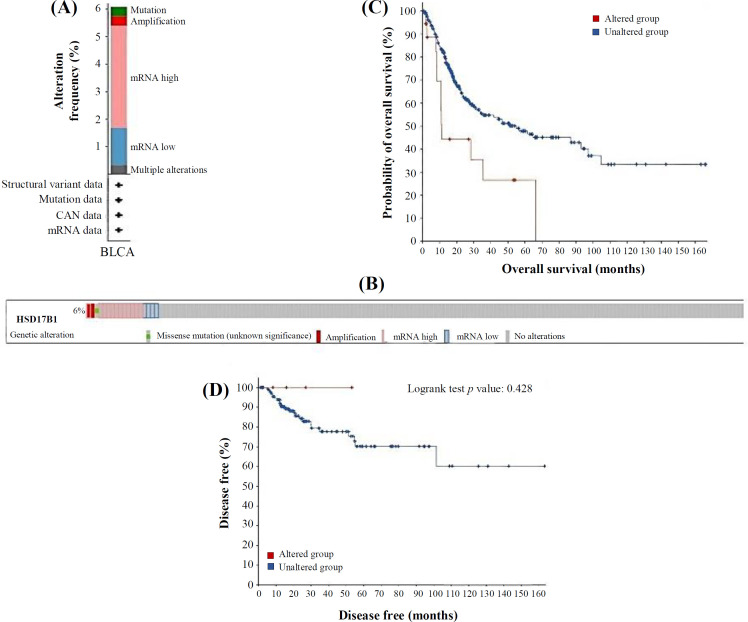
Genetic alterations in *HSD17B1* and their relationships with OS and DFS in BLCA patients (cBioPortal). (A) A list of *HSD17B1* mutations; (B) an OncoPrint visual a brief of alterations to the *HSD17B1* gene; (C) Kaplan-Meier plots contrasting patients' OS with and without an *HSD17B1* gene alteration; (D) Kaplan-Meier plots contrasting cases' DFS with and without an *HSD17B1* gene alteration

Genetic alterations are known to cause changes in gene expression and functions. We investigated whether the changes in *HSD17B1* mRNA expression were attributed to *HSD17B1* genetic alteration. The genetic

alteration of *HSD17B1* in BLCA was examined using cBioPortal. A total of 474 samples from BLCA MSK-TCGA-2020 database were studied. The genetic alteration rate of *HSD17B1* was detected only in 6% of the BLCA patients, in which the most frequent mutation resulting to increase in *HSD17B1* mRNA levels (Fig. 3A and 3B). Analysis of the Kaplan-Meier survival curves and a log-rank test indicated no significant variations in survival rates and disease-free intervals between the compared groups (Fig. 3C and 3D).

**Enrichment analysis of** ***HSD17B1*** **similar to expressed genes**

**Fig. 4:**
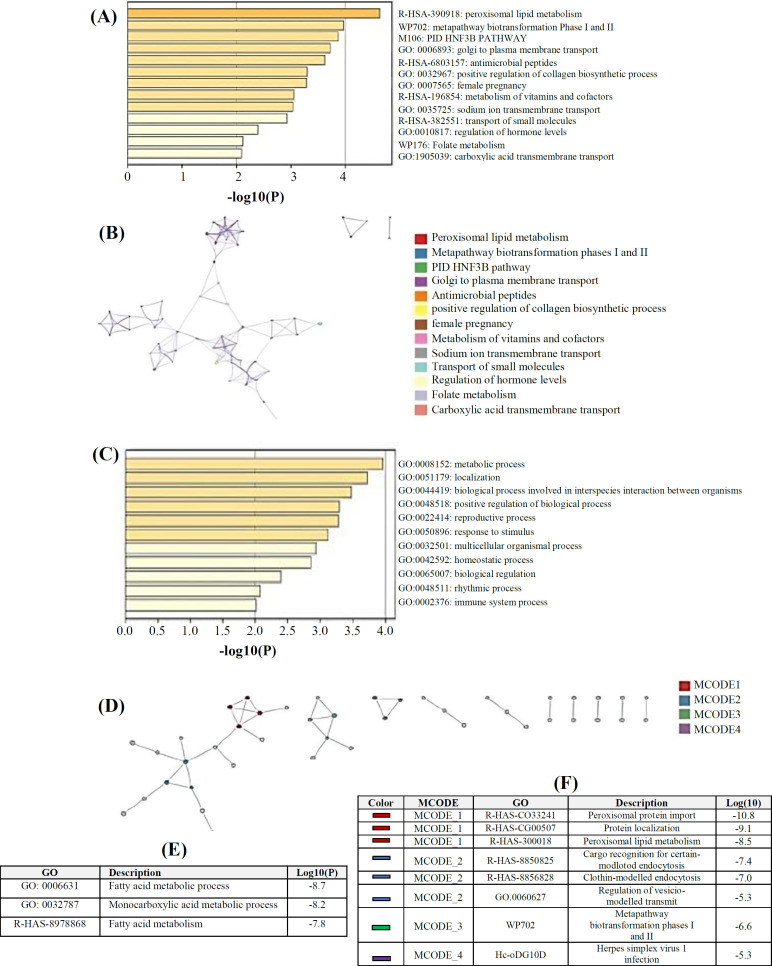
The enrichment analysis of HSD17B1 and its related genes in BLCA using Metascape. (A) A heatmap showing the enriched GO terms, where the p values for each term are color-coded; (B) network visualization of GO-enriched phrases, where terms with a greater number of genes tend to have a more significant p value; (C) A heatmap displaying enriched KEGG terms, color-coded by p values; (D) network visualization of KEGG-enriched terms, where terms with more genes typically tend to have a more significant p value; (E) top three functions enriched by physical interactions; (F) analysis of the functional enrichment of four MCODE components independently

The GEPIA database was used to identify the top 400 genes similar to *HSD17B1*. Metascape enrichment analysis was conducted to generate predictions of the functional roles of the *HSD17B1* similar genes. The top 20 GO enrichment items were divided into the biological processes, cellular components, and molecular functions categories (Fig. 4A and 4B and Table S1 and S2). The biological processes of *HSD17B1* and similar gene enrichment exhibited the metabolic processes, positively regulated biological processes, multicellular processes, and its other biological processes (Fig. 4C). Furthermore, the molecular functions regulated by *HSD17B1* and its related genes were predominantly enriched in peroxisomal lipid metabolism, transport of small molecules, and metabolism of vitamins and cofactors. Moreover, protein-protein interaction enrichment analysis was performed to determine the cellular functions of *HSD17B1*. Enrichment analysis of processes abd pathways was specifically conducted for each MCODE component using the MCODE algorithm (Fig. 4D). From the networks, we could find that the top three functions involved in the physical interactions were the fatty acid metabolic process, monocarboxylic acid metabolic process, and fatty acid metabolism (Fig. 4E and 4F).

**Fig. 5:**
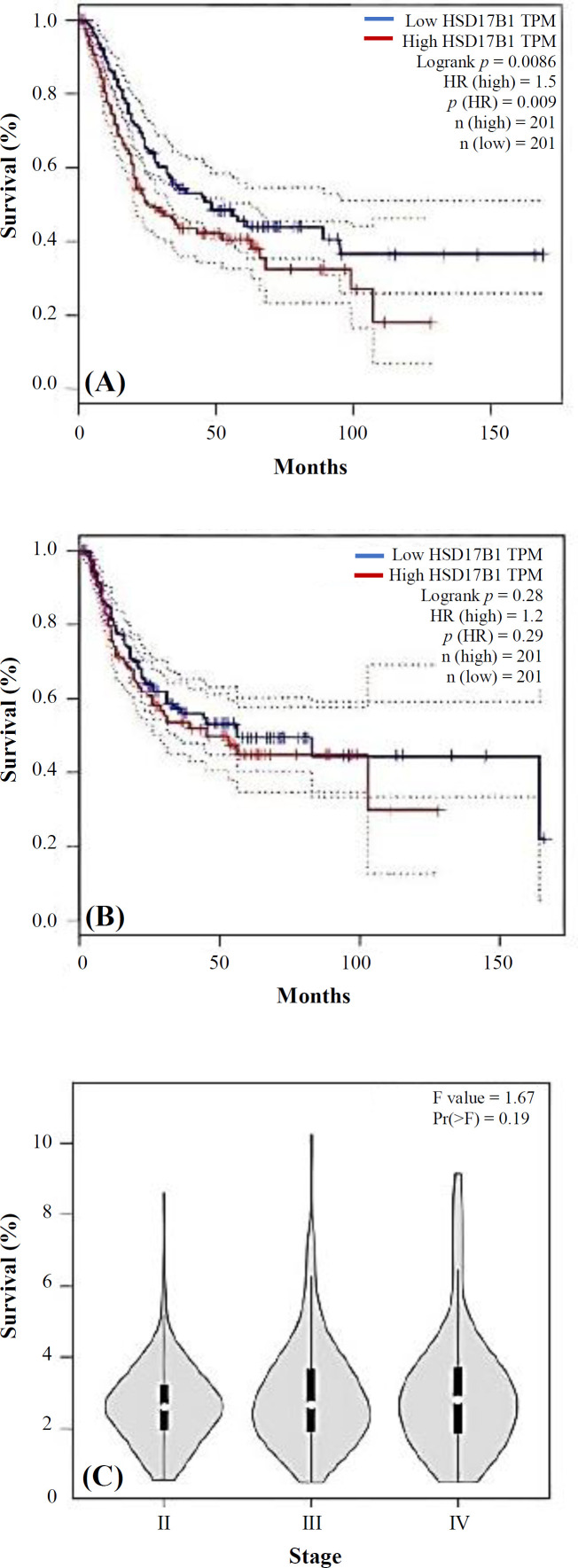
(A) Survival analyses of HSD17B1 gene (GEPIA); (B) DFS; (C) HSD17B1 stage plots

**Association between** ***HSD17B1*** **mRNA expression level and OS and DFS in BLCA patients**

We performed a survival analysis using the GEPIA database to assess the potential of *HSD17B1* as a prognostic marker in BLCA patients. The survival curves are shown in Figure 5. The *HSD17B1* expression gene was significantly associated with a reduced OS rate (*p* = 0.0086). Also, the DFS of BLCA patients was evaluated, and no statistically significant difference was observed (*p* = 0.28). We found an association between the level of *HSD17B1* gene expression and the grade of the tumor and demonstrated that the gene expression levels increased with elevating the tumor grade. **Methylation function in** ***HSD17B1*** **expression**

**Fig. 6:**
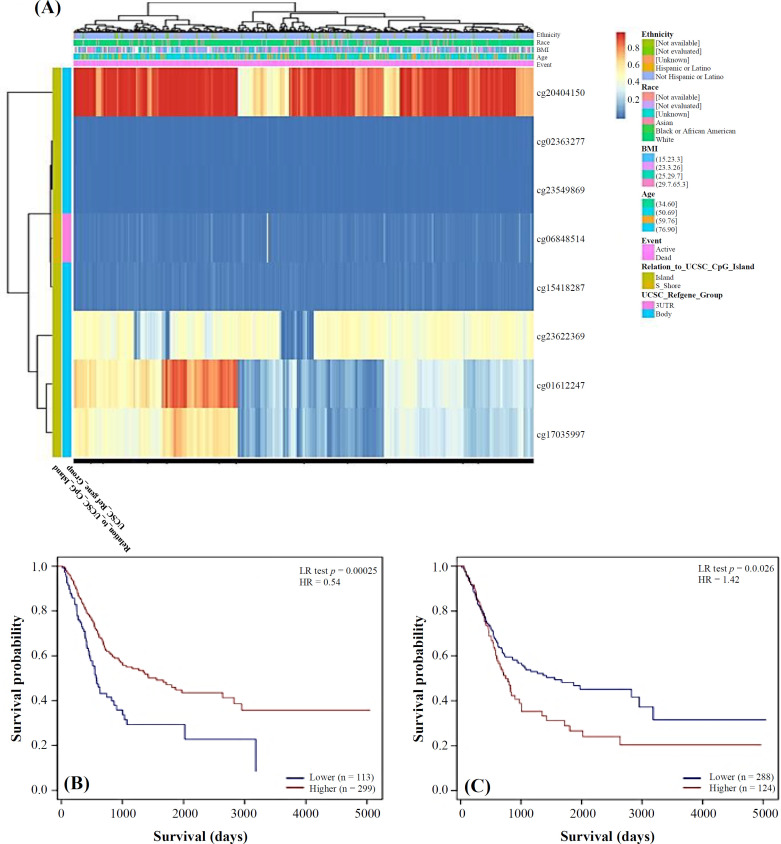
Association of DNA methylation levels in the *HSD17B1* gene with the prognosis of BLCA patients. (A) Heatmap DNA methylation; (B and C) CpG islands, cg20404150 and cg15418287, respectively. Methylation levels are significantly associated with  *p* < 0.05

We examined the DNA methylation level of *HSD17B1* and evaluated the predictive significance of its CpG islands using the MethSurv tool. Based on our analysis, of eight methylated CpG islands, two specific CpG islands, cg20404150 and cg15418287 exhibited an elevated DNA methylation level (Fig. 6 and Table S3). Furthermore, the level of methylation in both CpG islands were significantly associated with *HSD17B1* DNA methylation, with a *p* value of < 0.05 (Fig. 6B and 6C). Elevated levels of *HSD17B1* methylation in the two CpG islands, particularly cg20404150, were associated with poorer OS of BLCA patients, as compared to individuals with lower level of *HSD17B1* CPG methylation.

***Correlation between HSD17B1 expression level and immune-infiltrating cells***

 We utilized the TIMER to examine the presence of immune infiltrating cells associated with *HSD17B1*. The gene model was effectively employed to analyze the rate of tumor infiltration in cases exhibiting tumors with diverse immune cell types. Immune surveillance is commonly recognized as a significant determinant of the prognosis for various types of cancer. A total of 41 immune infiltration cell types were analyzed in the sample, with a cutoff *p* ≤ 0.05 (Fig. S2). Based on the analysis of immune cell differentiation, we determined that the levels of T cell CD8^+^ EPIC and mast cell resting CIBERSORT were significantly higher than other immune cells. Our results indicated that the increased expression of *HSD17B1* was strongly correlated with higher levels of immune infiltration cells, including mast cell resting CIBERSORT-ABS, T cell CD4^+^, , NK cell EPIC, NK cell resting CIBERSORT, and CD4^+^ cell resting_CIBERSO-ABS. Conversely, there was a significant negative correlation between *HSD17B1* expression and other immune infiltration cells, such as T cell CD4^+^ Central memory_XCELL, T cell CDA8+_CIBERSO-ABS, and Mast cell resting CIBERSORT (Fig. S2). Several immune-infiltrating cells, including mast cell resting CIBERSORT-ABS, demonstrated to serve as a tumor-associated biomarker gene with the potential to have significant effects on the immunological environment.

**Verifying the Expression of** ***HSD17B1*** **by qRT-PCR in Vitro**

**Fig. 7:**
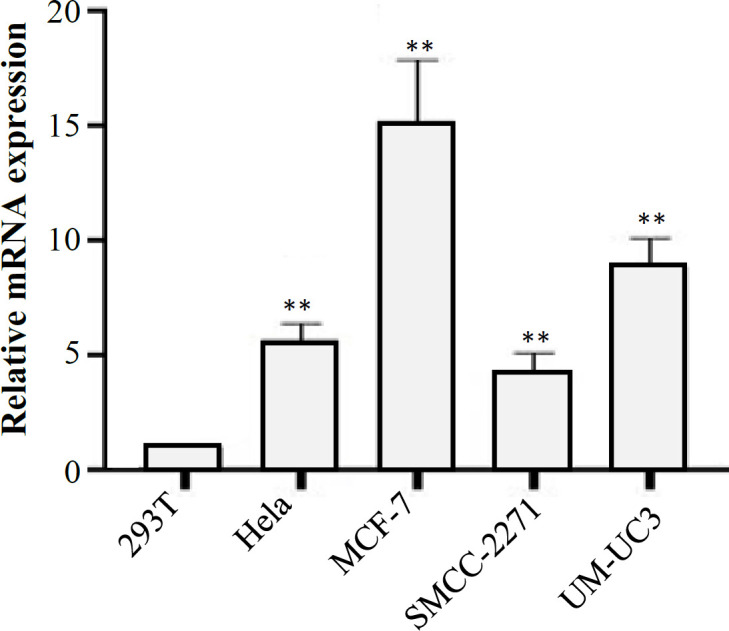
The relative mRNA expression levels of *HSD17B1* in five different cell lines, namely 293T, HeLa, MCF, SMMC-2271, and UM-UC3 using qRT-PCR (^**^p < 0.01)

We finally verified *HSD17B1* expression level in various tumor cells by qRT-PCR analysis. *HSD17B1* was significantly upregulated in MCF-7, UM-UC3, HeLa, and SMMC-2271 compared to 293T cells (Fig. 7). Similarly, according to the TCGA-UALCAN data, we also showed that *HSD17B1* expression level in cancer cells were more upregulated than the normal cells. (Fig. 1).

## DISCUSSION

 novel approaches may contribute to address these challenges and improve outcomes for BLCA patients. Therefore, it is essential to identify novel prognostic biomarkers and therapeutic interventions for BLCA to improve patient prognosis.

Cancer cells have been documented to alter cellular metabolism and energy management. Lipid metabolism regulates various biological functions, including cell growth, proliferation, differentiation, survival, apoptosis, inflammation, motility, membrane stability, chemotherapeutic responses, and drug resistance^[^^14^^]^. Reprogramming lipid metabolism has been demonstrated to be vital for supplying energy, macromolecules for membrane synthesis, and lipid signals as cancer grows^[^^15^^]^. In contrast, cancer is associated with severe metabolic alterations, one of which is dysregulation of lipid metabolism. Fatty acids, cholesterol, and phospholipids are some of the most prevalent lipid with functions such as energy sources, signaling molecules, and a supply of components for the synthesis of cell membranes^[^^16^^]^. 

Prior research has revealed that tumor tissues necessitate a high amount of lipid metabolism to support the requirements for processes such as membrane synthesis, energy storage, and signal transmission. Moreover, in lipid metabolism, fatty acid synthesis and the valeric acid pathway are strongly linked to cancer cell growth, differentiation, migration, and invasion^[^^17^^]^. Recently, scientific research has shown that all metabolic pathways, such as glucose, lipids, amino acids, and nucleotides, could serve as potential prognostic markers for BLCA. Our research also aimed to find a specific gene or gene expression associated with the consequences of the disease. In most cases, these gene expression products often refer to the etiology of various malignancies^[^^18^^]^.

In the present study, we hypothesized that *HSD17B1* may be linked to carcinogenesis and cancer progression, and this gene may play vital roles in BLCA by activating or inhibiting metabolism-related pathways, hence influencing the chemicals and energy required for tumor cell growth and reproduction. Our findings suggested that mRNA and protein expression of *HSD17B1* were significantly higher in cancer than in normal samples (Fig. 2). As a result, steroid hormones play an essential role in determining the lipid content of exposed tissues and HSD17Bs. The expression of genes, including *HSD17B1*, can be regulated by various factors such as transcription factors, epigenetic modifications, and signaling pathways. Changes in the regulation of the *HSD17B1* gene could lead to variations in protein expression levels^[^^19^^]^. High-grade urothelial carcinoma is often associated with genetic mutations. Mutation in genes involving in hormone metabolism, e.g. *HSD17B1*, could affect the expression of the corresponding protein. Mutations may result in upregulated or downregulated protein expression^[^^20^^]^. Research indicating their role in different cancer types is growing, and the expression pattern of HSD17Bs in cancer is considerably different from that in healthy tissue^[^^6^^,^^21^^]^. However, a significant association has been observed in the mRNA expression level of *HSD17B1* among cancer patients at stages 2, 3, and 4 compared to normal patients.

This current study investigated the genetic alteration of *HSD17B1* in BLCA and its association with the OS and DFS of BLCA. Our investigation revealed that the prevalence of genetic changes in *HSD17B1* among BLCA patients was only 6%, primarily characterized by elevated *HSD17B1* mRNA levels. Consistent with our findings, a prior investigation has demonstrated that changes in lipid metabolism are linked to the development of BLCA^[^^22^^]^, and this behavior may be due to cellular estrogen metabolism, resulting in increased synthesis of active estrogens^[^^23^^]^. Herein, we performed an enrichment analysis on pathways and processes for each MCODE component detected by the algorithm with three functions, i.e. the fatty acid metabolic process, the monocarboxylic acid metabolic process, and fatty acid metabolism, which have been previously enhanced by physical interactions. In prior functional analyses, lipid-related genes exhibited a significant association with the peroxisome proliferator-activated receptor signaling pathway, fatty acid metabolism, and the AMP-activated protein kinase signaling pathway^[^^24^^]^. 

When comparing CpG sites in BLCA samples, we observed that two specific CpG islands, cg20404150 and cg15418287, exhibited elevated level of DNA methylation. This raise was found to be correlated with the prognosis of higher levels of *HSD17B1* methylation in these two specific CpG islands, especially cg20404150. Moreover, it is associated with a lower OS rate in BLCA patients, as compared to those with lower levels of *HSD17B1* CPG methylation. A prior study indicated a connection between the *HSD17B1 *expression and DNA methylation in cancer^[^^25^^]^. This outcome could arise from the occurrence of DNA hypermethylation and histone modifications, which are key factors in epigenetic regulation and have a crucial role in the suppression of genes in all types of malignancies^[^^26^^]^. Consistent with our research findings, a previous study has demonstrated that human *HSD17B1* is mainly expressed in tissues that produce estrogen, particularly the ovary tissue and the placenta^[37]^. However, *HSD17B1 *can also be detected at lower levels in peripheral estrogen target tissues, such as the breast^[^^28^^]^ and endometrium^[^^29^^]^. Furthermore, the presence of *HSD17B1* has been confirmed in non-small cell lung cancer cell lines that facilitates the conversion of E1 to E2, suggesting that this gene acts as a mediator in this conversion process^[^^30^^,^^31^^]^. In a mostly post-menopausal group of patients, those who expressed *HSD17B1* mRNA or protein had notably lower overall and DFS rates than the other patients^[^^32^^]^. Conversely, our study revealed that the elevated levels of *HSD17B1* mRNA were linked to lower OS.

In this study, *HSD17B1* could be used to predict BLCA patient survival. Moreover, it could serves as a reliable additional indicator for BLCA. Utilizing *HSD17B1* in conjunction with other well-established biomarkers would significantly improve the early detection and prognosis of BLCA.

## CONCLUSION

Our findings demonstrate a significant association between the overexpression of *HSD17B1* and both the clinical stages and pathological grades of tumors in patients with BLCA. Furthermore, there was a positive correlation between increased expression of *HSD17B1* mRNA and OS. *HSD17B1* also showed to be a potential biomarker for predicting the prognosis of BLCA. More studies with larger sample sizes are needed to prove our findings, and more related research is required to investigate the intricate mechanism underlying *HSD17B1* expression and BLCA.

## DECLARATIONS

### Acknowledgments

 Authors declare that they do not have used any AI technology in generation of current research work.

### Ethical approval

 Not applicable

### Consent to participate

Not applicable

### Consent for publication

All authors reviewed the results and approved the final version of the manuscript.

### Authors’ contributions

AA: conceived and designed the experiments, performed the experiments, prepared figures and tables, and drafted manuscript preparation and visualization. MA: analyzed the data and interpreted of results. HC, YX, and JS: critically revised and edited the manuscript; PS: designed the conception and approved the final version of manuscript.

### Data availability

All data generated or analyzed during this study are included in this published article.

### Competing interests

The authors declare that they have no competing interests.

### Funding

The study received support from a grant provided by the Development Project of Qinghai Provincial Key Laboratory (2022-ZJ-Y18).

### Supplementary information

The online version contains supplementary material. 
